# Efficacy information influences intention to take COVID‐19 vaccine

**DOI:** 10.1111/bjhp.12546

**Published:** 2021-07-11

**Authors:** Colin J. Davis, Matt Golding, Ryan McKay

**Affiliations:** ^1^ School of Psychological Science University of Bristol; ^2^ Rubber Republic Bristol UK; ^3^ Department of Psychology Royal Holloway University of London UK

**Keywords:** COVID‐19, vaccines, efficacy, health communication, protection motivation theory

## Abstract

**Objectives:**

A successful response to the COVID‐19 pandemic requires achieving high levels of vaccine uptake. We tested whether directly contrasting the high efficacy of COVID‐19 vaccines with the lower efficacy of the annual flu vaccine would increase intentions to take a COVID‐19 vaccine.

**Design:**

A pre‐registered online study of 481 participants compared four information conditions: (1) no information; (2) COVID‐19 Vaccine Information Only; and COVID‐19 Vaccine Information combined with flu vaccine information suggesting either (3) 60% efficacy or (4) 40% efficacy; we measured COVID‐19 and flu vaccine intentions along with several other vaccine‐related variables.

**Methods:**

The Prolific platform was used to recruit 481 UK participants (64% female; aged between 18 and 85 years) who had been pre‐screened to have intermediate levels of vaccine hesitancy. After reading a short text (~200 words) about COVID‐19 vaccines, participants were asked about their vaccination intentions.

**Results:**

Providing information about the safety and efficacy of the new COVID‐19 vaccines resulted in vaccination intentions that were, on average, 0.39 standard deviations (*SDs*) higher than those in the no information condition; providing the same COVID vaccine efficacy information in the context of information about flu vaccine efficacy resulted in a further significant increase in vaccination intentions that were 0.68 *SD* higher than those in the no information condition. This positive contrast effect for the COVID‐19 vaccine was not associated with reduced flu vaccine intentions.

**Conclusions:**

Vaccination intentions can be strengthened through a simple messaging intervention that utilizes context effects to increase perceived response efficacy.

**Statement of Contribution**

***What*** ***is already known on this subject?***

- Very high levels of COVID‐19 vaccine uptake are required to attain herd immunity. Previous experience and recent surveys suggest that achieving such high levels of uptake will be challenging.
- Protection motivation theory predicts that high uptake requires both high threat appraisal and high coping appraisal. An important aspect of coping appraisal is response efficacy.
- Previous studies suggest that response efficacy is a strong predictor of vaccine intentions and behaviour, but to date we lack interventions that reliably boost response efficacy.

***What*** ***does this study add?***

- The results show that providing information about the safety and efficacy of the new COVID‐19 vaccines results in higher vaccination intentions than among those who do not receive this information.
- Providing COVID vaccine efficacy information in the context of information about flu vaccine efficacy results in even stronger COVID‐19 vaccination intentions, without undermining flu vaccine intentions.
- These findings support the role of response efficacy and suggest that perceived response efficacy can be boosted via a simple intervention that draws on cognitive theory.

## Background

Along with the introduction of safe drinking water, vaccination is the greatest success of modern public health, saving millions of lives every year. In addition to the direct protection offered to those individuals who receive a vaccine, vaccination indirectly protects those around them by reducing the spread of disease. The latest vaccines to be introduced are those that provide protection against COVID‐19, the disease caused by the SARS‐CoV‐2 virus. The speed with which these vaccines have been developed is an impressive achievement, given that vaccines typically take decades to develop and get to the point of approval.

### Vaccine uptake

Nevertheless, it is one thing to have an effective vaccine and another thing to have a vaccinated population. The minimum level of coverage of a population required to attain herd immunity against a virus depends on the basic reproductive number *R*_0_ of that virus.^1^ In the case of COVID‐19, this coverage was initially estimated at around 70% (Kwok, Lai, Wei, Wong, & Tang, 2020). However, the emergence of new SARS‐CoV‐2 variants of higher transmissibility implies that an even higher level of coverage (>80%) may be required to reach herd immunity. In practice, achieving such a high coverage requires an even greater proportion of the population to be vaccinated (or infected), given that vaccine efficacy is <100%.

It is not easy to achieve such high levels of vaccine uptake. For example, although the seasonal flu vaccine is recommended for all individuals above the age of 6 months, levels of uptake are often low. To illustrate, among US adults, flu vaccine coverage was only 37.1% during the 2017–2018 season, a year in which the flu was associated with particularly high levels of illnesses, hospitalizations, and deaths (the US Center for Disease Control [CDC] estimated there were 79,400 deaths overall in the United States, including 10,300 deaths among working age adults; CDC, 2018). In such cases, the low level of uptake does not reflect a lack of vaccine or major barriers to the access to vaccines, but rather *vaccine hesitancy*, which the World Health Organization (WHO) defines as ‘delay in acceptance or refusal of vaccines despite availability of vaccination services’ (WHO, 2014). The level of vaccine hesitancy and the reasons for it can vary by sub‐population (Ozawa et al., 2019; Quinn, Jamison, Musa, Hilyard, & Freimuth, 2016).

Some of the problems with vaccine uptake can be tackled by economic and policy measures, such as broadening the scope of health insurance and framing vaccination as the default option. However, vaccine uptake is also affected by people’s attitudes and beliefs (e.g., Murphy et al., 2021). Behavioural scientists have a role to play to better understand the factors affecting vaccine hesitancy, and to explore how we can influence behaviour to boost vaccine uptake.

### Types of vaccine hesitancy

Though media representations often depict polarized pro‐ and anti‐vaccine positions, there is a continuum of attitudes to vaccines that ranges from outright refusal to complete acceptance (e.g., Quinn et al., 2016). Prior to the widespread rollout of COVID‐19 vaccination, surveys in the United Kingdom and other countries indicated that most adults expressed an intention to take a COVID‐19 vaccine if it were offered (Detoc et al., 2020; Sherman et al., 2020); as we describe below, we observed a similar trend in our own survey. Nevertheless, there remains a significant minority who do not express a definite intention to take a COVID‐19 vaccine if it is offered to them. It is useful to distinguish two groups within this minority (e.g., Pierre, 2020). On the one hand, there are those who are extremely hesitant, vulnerable to conspiracy theories, and who may fall into the category of ‘anti‐vaxxers’. Tackling vaccine hesitancy in this group may depend on techniques for reducing the impact of misinformation (Lewandowsky, Ecker, Seifert, Schwarz, & Cook, 2012).

On the other hand, there are those who do not reject all vaccines, but who are cautious about the COVID‐19 vaccine. Such individuals may not be inclined to take a vaccine simply because it is recommended by an authority, preferring to collect further evidence before making their decision. It is this group who are the focus of the present study. Individuals in this group have ambivalent or even moderately favourable attitudes towards the COVID‐19 vaccine, but are at relatively high risk of not ultimately being vaccinated. We can refer to this group as the ‘cautious middle’ or ‘fencesitters’ (Gust et al., 2005).

### Protection motivation theory

Theories from health psychology provide a framework for describing the factors underlying vaccine hesitancy and may inform interventions to increase vaccine uptake. One theory that has helped to understand vaccine behaviour is Protection motivation theory (Maddux & Rogers, 1983). According to this theory, people’s motivation to protect themselves is a function of their *threat appraisal*, that is, their perception of the health threat, and their *coping appraisal*, that is, their perception of what can be done to reduce or prevent that threat. The theory predicts that high uptake requires both high threat appraisal and high coping appraisal.

#### Threat appraisal

Appraisal of a health threat involves considering both the severity of that threat and one’s personal vulnerability to it. In accord with protection motivation theory, vaccine intentions are strongly related to risk perception (e.g., Brewer et al., 2007; Ernsting, Lippke, Schwarzer, & Schneider, 2011; Freimuth, Jamison, An, Hancock, & Quinn, 2017). This relationship can explain the lower levels of influenza vaccine uptake amongst younger people, who tend to be less worried about catching the flu, despite being at high risk for influenza morbidity (Bednarczyk et al., 2015). In the case of COVID‐19, there is a relatively high degree of concern about the risks associated with the disease, both with respect to personal susceptibility to being infected and the consequences of becoming infected (Dryhurst et al., 2020). These high levels of concern can be predicted to promote high vaccination uptake.

#### Coping appraisal

An individual’s coping appraisal involves considering self‐efficacy (one’s own ability to take the necessary preventative action), response efficacy (how likely the recommended behaviour is to alleviate the threat), and response costs (barriers to performance of the behaviour, such as anticipated side effects of vaccination). Previous research has found evidence for all three of these factors in predicting vaccine behaviour (e.g., Ehrenstein et al., 2010; Ling, Kothe, & Mullan, 2019; Quinn et al., 2016).

Several studies have offered evidence for the influence of perceived response costs. Parents who delay or refuse immunization for their children are significantly less likely to believe that vaccines are safe (Gust et al., 2004). Likewise, parents with stronger beliefs that vaccination is unhealthy and can harm the immune system are more likely to refuse the combined Measles, Mumps, and Rubella (MMR) vaccine for their children (Flynn & Ogden, 2004). These studies point to the importance of assuaging safety concerns. Indeed, safety scares can lead to substantial drops in seasonal influenza vaccination (e.g., Rosselli, Martini, Bragazzi, & Watad, 2017). However, not all studies have observed clear effects of perceived response costs (Camerini, Diviani, Fadda, & Schulz, 2019; Ling et al., 2019).

In the case of vaccination, self‐efficacy refers to an individual’s perception of their own ability to get vaccinated while response efficacy reflects an individual’s perception of the efficacy of the vaccine, that is, how effectively it will protect them from the disease. Self‐efficacy for taking a vaccine is related to the availability, affordability, and accessibility of the vaccine. Uptake is lower if these factors are perceived to pose an obstacle (e.g., Ling et al., 2019; Quinn et al., 2016). Response efficacy is typically measured using items such as ‘I believe if I receive the flu vaccine, I will be less likely to get the flu’. Responses to such items are strongly positively correlated with vaccine intentions and behaviour (Ernsting et al., 2011; Ling et al., 2019; Pareek & Pattison, 2000; for more examples in the case of vaccination against pandemic influenza, see the systematic review by Bish, Yardley, Nicoll, and Michie, 2011). Systematic reviews of the factors influencing seasonal flu vaccination have consistently found that uptake was associated with higher perceived effectiveness (Chapman & Coups, 1999; Yeung, Lam, & Coker, 2016). Indeed, perceived response efficacy may be the best predictor of vaccine intentions and behaviour. For example, Pareek and Pattison (2000) found that vaccine outcome beliefs explained 77% of the variance in mothers’ MMR vaccine intentions. Similarly, a mediation analysis by Ernsting et al. (2011) suggested that the influence of risk perception and negative outcome expectancies was limited to an indirect effect on vaccination behaviour via the formation of intentions, whereas positive outcome expectancies had both an indirect and a direct effect on vaccine behaviour.

### Designing an intervention to boost vaccine uptake

Given the need to achieve very high levels of vaccine uptake so as to attain herd immunity, the question of how to boost uptake of COVID‐19 vaccines is a pressing concern. Protection motivation theory and the sample of relevant evidence reviewed above suggest potential areas for intervention. In view of the already high levels of threat associated with COVID‐19, the most promising interventions for increasing vaccine uptake are those that seek to influence coping appraisal. Of these, self‐efficacy should be targeted, but in practice this is most likely to involve policy interventions, for example, making the COVID‐19 vaccine freely available, placing vaccination centres in highly populated areas, and so on. The remaining two aspects of coping appraisal, response efficacy and response costs, are both potential targets for intervention, with the former likely to be particularly important.

Beliefs in the safety and efficacy of vaccines are often grouped together under the heading *vaccine confidence*. The WHO has recognized the importance of monitoring vaccine confidence and its role in vaccine uptake (Salmon & Dudley, 2020). To date, though, interventions to increase vaccine confidence have shown limited success. A review by Brewer, Chapman, Rothman, Leask, and Kempe (2017) bemoaned the absence of effective interventions to boost confidence in individuals for whom it is not high, noting that, ‘we do not know how to reliably increase vaccine confidence. Interventions to increase confidence through persuasion and education have had no appreciable or reliable effect on vaccination coverage’ (p. 163).

A consideration that may be relevant to the perceived response efficacy of COVID‐19 vaccines is the fact that the objective efficacy levels are extremely high. When our study was conducted, the outcome of Phase 3 trials had been reported for two COVID‐19 vaccines: the Pfizer vaccine had an efficacy of 95% (Pfizer‐Press‐Release, 2020) and the Moderna vaccine had an efficacy of 94% (Moderna‐Press‐Release, 2020). This very high level of efficacy can be contrasted with that of more familiar vaccines, notably the annual flu vaccine. The typical seasonal flu vaccine has an efficacy no higher than 60% (Cohen, 2017), and the mean for the 15 flu seasons from 2004–2005 to 2018–2019 was 40% (CDC, 2019). Thus, the new COVID‐19 vaccines have a much higher efficacy than the flu vaccines that have been used for many years.

Such a contrast is not merely academic – it may be relevant to the way people consider vaccines and the way vaccine messaging is presented. Explicitly comparing the efficacy of the novel COVID‐19 vaccines with the more familiar flu vaccine focuses the individual’s attention on efficacy as the key evaluative dimension (Tversky, 1977), drawing attention away from other dimensions (e.g., the novelty of the vaccine). Given the focus on efficacy, and the context provided by the much lower efficacy of the flu vaccine, we can expect a contrast effect (Schwartz & Bless, 1992), amplifying the favourable perception of the COVID‐19 vaccine. These considerations led us to predict that providing participants with knowledge about the relative efficacy of the COVID‐19 and flu vaccines would increase the stated intention to take the COVID‐19 vaccine.

This potential benefit notwithstanding, a potential drawback of the efficacy comparison is the possibility that it could undermine the perceived efficacy of the flu vaccine and lead to a reduced uptake of this vaccine. This would be a very negative outcome, as even a very low efficacy (e.g., 20%) flu vaccine has the potential to avert millions of infections, hundreds of thousands of hospitalizations, and tens of thousands of deaths, provided coverage is high (Sah, Medlock, Fitzpatrick, Singer, & Galvani, 2018). It would not be rational to reduce one’s confidence in the flu vaccine based on learning about the higher efficacy of a vaccine developed to tackle a different disease. However, if people tend to overestimate the efficacy of the flu vaccine, this overestimation may be made salient through the provision of information about the true efficacy.

### The current study

The primary goals of the present study were to test whether this positive contrast effect could increase intentions to take a COVID‐19 vaccine, and to check whether there is any negative contrast effect on intentions to take an annual flu vaccine. We also sought to (1) determine what the estimated efficacy of the flu vaccine is, and whether this systematically overestimates the true efficacy; and (2) investigate the effect of providing information about flu vaccine efficacy on both perceived efficacy and intention to take the flu vaccine.

## Method

### Pre‐screening

Based on a prior experiment, we expected the distribution of COVID‐19 vaccine intentions to be strongly skewed towards high levels of acceptance. That experiment showed that, overall, vaccine information did not increase vaccine intentions, due to an apparent ceiling effect, but that there was evidence of an effect of vaccine information on those with intermediate levels of vaccine intentions. We therefore pre‐registered our intention to examine this effect on a sample of participants who had been pre‐screened to belong to this ‘cautious middle’ demographic.

For the pre‐screening, we asked participants to respond to the statement, ‘I intend to take a COVID vaccine’ on a 7‐point scale from *strongly disagree* to *strongly agree*. Participants were recruited via the Prolific platform. They had already been pre‐screened to be adult residents of the United Kingdom who had English as their first language and who had no (self‐reported) language disorders and had not been infected with COVID‐19 (this was a screening variable made available by the Prolific platform, but our experiment also included a question about infection to exclude participants who had been infected subsequent to the Prolific screening question). We obtained responses from 2000 participants on 11 December 2020 and a further 400 participants on 15 December 2020. The distribution of responses is shown in Table 1. As can be seen, most respondents showed strong vaccine intentions. There were 1,021 (23.2%) who fell into the cautious middle category by virtue of responding ‘Somewhat agree’, ‘Neither agree nor disagree’ or ‘Somewhat disagree’.

**Table 1:** Distribution of responses in pre‐screening question (‘I intend to take a COVID vaccine’)

Response	*n*	%
Strongly agree	1,782	40.5
Agree	1,069	24.3
Somewhat agree	507	11.5
Neither agree nor disagre	300	6.8
Somewhat disagree	214	4.9
Disagree	239	5.4
Strongly disagre	289	6.6

### Participants

The experiment was advertised to these 1,021 individuals, and data collection took place between 12 December and 17 December 2020. Following our pre‐registered analysis plan, 81 participants were excluded from the analysis. Of these, 41 had already tested positive for COVID‐19 (or believed that they had had COVID‐19) and 40 failed one or both attention checks. These participants were replaced, so that we ultimately had *n* = 100 participants in each of the four conditions.^2^ Of these, 64% were female, and one person reported their gender as Other. The age of participants ranged between 18 and 85, with a median of 36 and mean of 37.8 years.

### Stimuli and design

The experiment involved a single factor (information condition) between‐groups design. Participants were randomly assigned to one of four conditions: (1) no information (participants were asked questions about vaccines and their intentions without receiving any prior information); (2) COVID‐19 Vaccine Information Only (participants first received information about the efficacy and safety of COVID‐19 vaccines); (3) Flu Information – 60% (participants received the COVID‐19 Vaccine Information together with the information that flu vaccine efficacy has not exceeded about 60% in recent years); or (4) Flu Information – 40% (participants received the COVID‐19 Vaccine Information together with the information that the average flu vaccine efficacy in recent years has been 40%). The goal of the manipulation of flu vaccine efficacy information was to gauge how sensitive participants were to variations in efficacy. The specific values chosen for conditions (3) and (4) were consistent with different ways of summarizing evidence, and the difference between the values seemed sufficiently large that it was feasible that participants might distinguish them.

The full set of stimuli for this experiment, as well as our pre‐registered hypotheses and analysis plan and the raw data, can be found at https://osf.io/w4nmv/.

### Procedure

The study was self‐certified in accordance with the ethics procedure of Royal Holloway, University of London, and all participants provided their informed consent. Participants were presented with a short text (~200 words) about COVID‐19 vaccines, describing their safety and efficacy and the positive implications of widespread uptake (participants in the no information condition did not see this text). The full text is reproduced in the Appendix.

Following the text, participants were asked two questions about their vaccination intentions, five questions about vaccine efficacy (two of which elicited the percentages mentioned in the provided text and which thus served as an attention check), one question about their previous vaccination behaviour, one question about whether they had previously contracted COVID‐19, and two demographic questions.

#### Vaccination intentions

The first question asked, ‘If you were offered a COVID‐19 vaccine tomorrow, how likely is it that you would take the vaccine?’. The second question asked, ‘How likely is it that you will take the flu vaccine in the future?’. Responses to both questions were made using a slider representing an 11‐point scale from *not likely at all* to *very likely*. The responses to these questions are the two key dependent variables.

#### Perceived vaccine efficacy

This construct was measured using three items from Kim, Pjesivac, and Jin (2019; 2017, adapted from Witte, Meyer, & Martell, 2001). Participants were asked to indicate the degree to which they agreed with the following three statements on a scale of 1 (*strongly disagree*) to 7 (*strongly agree*): (1) ‘I believe the flu vaccine is effective in preventing the flu’; (2) ‘I believe if I receive the flu vaccine, I will be less likely to get the flu’; and (3) ‘I believe the flu vaccine works in preventing the flu’. The responses were averaged to form a single PVE measure.

#### Attention check

These two questions asked about the efficacy of the COVID‐19 and flu vaccines: ‘The text that you read mentioned the efficacy of (COVID‐19 vaccines)/(the annual flu vaccine). Please move the slider to roughly correspond to the efficacy level mentioned in the text’. These questions served as an attention check, to ensure that participants encoded the information that was critical to the experimental manipulation. Participants in the no information conditions did not see the flu vaccine version of this question (as they were not presented with any text about the efficacy of this vaccine). Instead, they saw the following question: ‘Please give your best guess of the average efficacy of the annual flu vaccine’. Responses to this question provide an index of the general population’s knowledge of the flu vaccine efficacy (we expected that this measure would be an overestimate of the true average efficacy).

Participants in the no information condition did not see the COVID‐19 vaccine efficacy attention check question (as they were not presented with any text about this vaccine’s efficacy). Instead, they saw the following text and question: ‘Vaccine efficacy is the percentage reduction of disease in a vaccinated group of people compared to an unvaccinated group, under the most favourable conditions. For example, a vaccine with 100% efficacy would protect everyone who received it from becoming sick. Based on what you have heard of COVID‐19 vaccines, what is your best guess of their efficacy?’ Responses to this question provide an index of the general population’s beliefs about COVID‐19 vaccine efficacy.

#### Vaccination behaviour

A single question asked, ‘Have you already taken a flu vaccine this year?’ (yes/no).

#### COVID‐19 infection

A single question asked, ‘Do you know or strongly believe that you have been infected by the virus that causes COVID‐19?’ (yes/no).

#### Demographic variables

Participants were asked to indicate their age and gender. The experiment took approximately 3 min in total.

## Results

Mean vaccine intentions and perceived flu vaccine efficacy for each condition are shown in Table 2.

**Table 2:** Means (and SDs) of perceived vaccine efficacy (PVE), flu vaccine intentions (FVI), and COVID‐19 vaccine intentions (C19VI) by information condition

Condition	*n*	PVE	FVI	C19VI
No information	100	5.09 (1.17)	5.27 (3.24)	4.61 (2.40)
COVID‐19 vaccine info only	100	5.15 (1.11)	5.78 (3.17)	5.53 (2.30)
Flu info – 60% efficacy	100	5.16 (1.07)	5.76 (3.34)	5.95 (2.64)
Flu info – 40% efficacy	100	5.12 (0.97)	5.84 (3.17)	6.26 (2.48)

### COVID‐19 vaccine intentions

**Figure 1:**
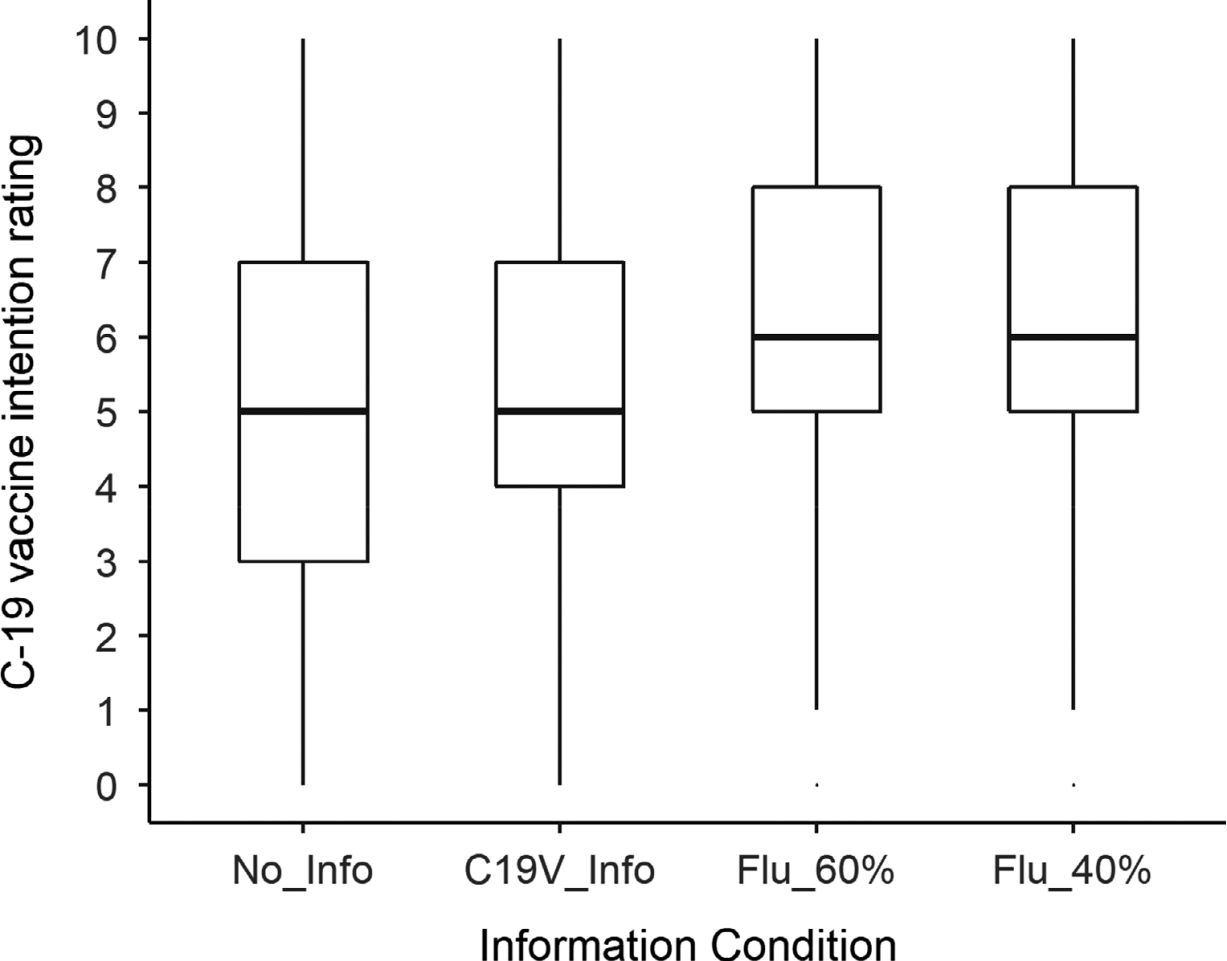
COVID‐19 vaccine intention as a function of information condition.

We pre‐registered three planned, directional (i.e., one‐tailed) *t*‐tests. A nominal alpha criterion of 0.05 was assumed for testing statistical significance; after Bonferroni correction, a conservative criterion of 0.05/3 = 0.017 was employed for testing these three tests. The first test showed that participants who received information about (only) COVID‐19 vaccines subsequently showed a stronger intention to take a COVID‐19 vaccine (mean = 5.53) than participants who did not receive any information (mean = 4.61), *t*(198) = 2.77, *p* = .003, *d* = 0.39. As can be seen in Figure 1, this effect resulted from a shrinking of the distribution in the COVID‐19 Vaccine Info Only condition, suggesting that the safety and efficacy information helped to assuage the concerns of some of the more hesitant participants, but did not greatly influence the participants who were already slightly more positive.

The second test showed that participants who received the 40% flu vaccine efficacy information subsequently showed a stronger intention to take a COVID‐19 vaccine (mean = 6.26) than participants who received COVID‐19 vaccine information but no flu vaccine information (mean = 5.53), *t*(197) = 2.16, *p* = .016, *d* = 0.31. As can be seen in Figure 1, this effect seems to have been driven by a shift in the distribution.

The third test compared participants who received flu vaccine efficacy information (describing either 40% or 60% efficacy) (mean = 6.10) with participants who received COVID‐19 vaccine information but no flu vaccine information (mean = 5.53). There was a numerical difference suggesting greater intention to take the COVID‐19 vaccine in the former group, but this did not quite attain significance after Bonferroni correction, *t*(218) = 1.96, *p* = .025, *d* = 0.24.

### Flu vaccine intentions

**Figure 2:**
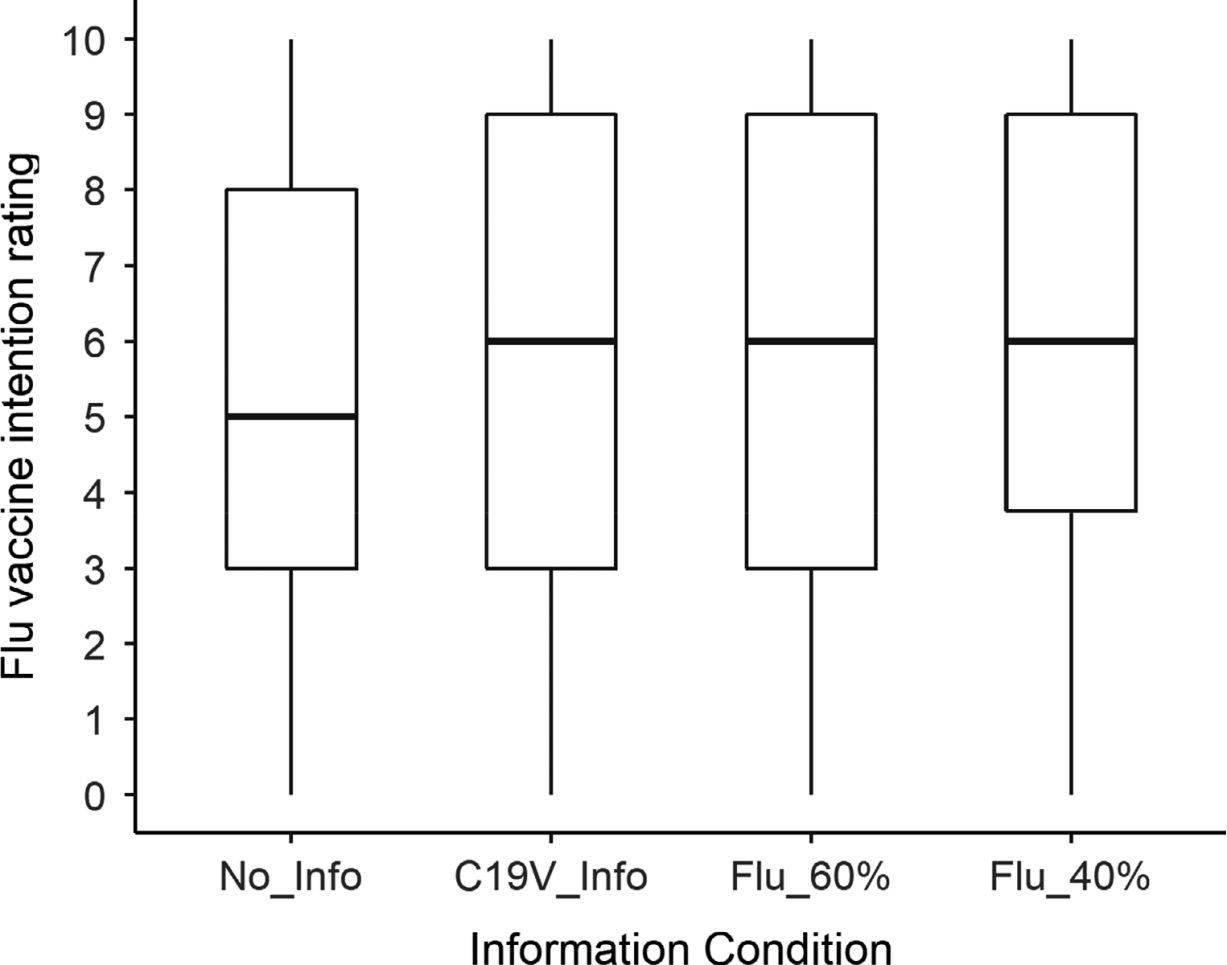
Flu vaccine intention as a function of information condition.

Mean flu vaccine intentions for the four information conditions are shown in Figure 2. We pre‐registered three planned, directional *t*‐tests concerning the impact of information on flu vaccine intentions. The first test indicated no difference in flu vaccine intentions between participants who received the 40% flu vaccine efficacy information (mean = 5.84) and those who did not receive any flu vaccine information (only the COVID‐19 vaccine information; mean = 5.78), *t*(198) = 0.13, *p* = .55.

The second test indicated no difference in flu vaccine intentions between participants who received flu vaccine efficacy information (describing either 40% or 60% efficacy; mean = 5.80) and those who did not receive any flu vaccine information (only the COVID‐19 vaccine information; mean = 5.78), *t*(203) = 0.05, *p* = .52.

The third test indicated no difference in flu vaccine intentions between participants who received the 40% flu vaccine efficacy information (mean = 5.84) and those who received the 60% flu vaccine efficacy information (mean = 5.76), *t*(197) = 0.17, *p* = .57.

Inspection of the means led us to conduct an additional test. This (two‐tailed) test compared flu vaccine intentions for those who received information (mean = 5.79) versus those who received no information (mean = 5.27). The difference was not significant, *t*(169) = 1.40, *p* = .16.

### Perceived flu vaccine efficacy

**Figure 3:**
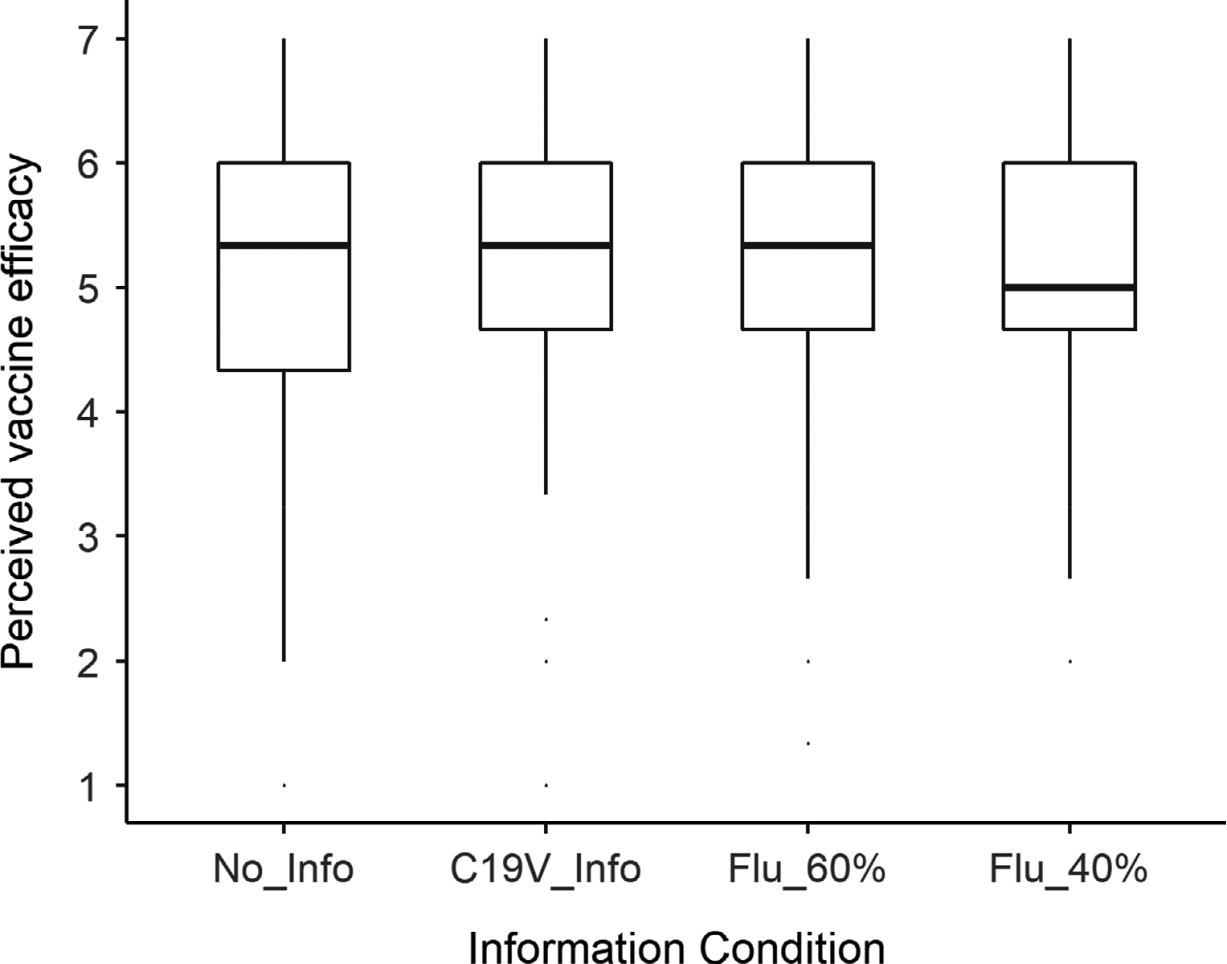
Mean perceived flu vaccine efficacy as a function of information condition.

The correlation between perceived flu vaccine efficacy and estimated vaccine efficacy was *r* = 0.52, which was significant (*p *< .001), but sufficiently smaller than 1 to suggest that these are distinct constructs. Figure 3 shows mean perceived flu vaccine efficacy for the four information conditions. As can be seen, there was little indication of any difference between conditions. This was confirmed by the pre‐registered planned tests. The first test indicated no difference in perceived flu vaccine efficacy between participants who received the 40% flu vaccine efficacy information (mean = 5.12) and those who did not receive any flu vaccine information (only the COVID‐19 vaccine information; mean = 5.15), *t*(195) = 0.23, *p* = .41.

The second test indicated no difference in perceived flu vaccine efficacy between participants who received flu vaccine efficacy information (mean = 5.14) and those who did not receive any flu vaccine information (only the COVID‐19 vaccine information; mean = 5.15), *t*(184) = 0.08, *p* = .47.

### Relationship between perceived flu vaccine efficacy and intentions

To explore the factors affecting flu vaccine intentions, we conducted a multiple regression with age, gender, and perceived flu vaccine efficacy, and (for the subset of participants who were asked to guess) estimated objective vaccine efficacy as predictors; the resulting model explained 26% of the variance in intentions (see Table 3). Most of this variance was accounted for perceived flu vaccine efficacy, which explained 25% of the variance intentions by itself (the zero‐order correlation was .50, i.e., greater PVE was associated with stronger intentions to take a flu vaccine). The only other significant predictor was gender, which reflected a small effect whereby males expressed weaker intentions to take up a flu vaccine than females. In particular, estimated objective vaccine efficacy was not a significant predictor.

**Table 3:** Multiple regression predicting flu vaccine intentions

Predictor	b	95% CI	*t*(194)	p
Intercept	−1.29	[−3.91, 1.33]	−0.97	.332
Age	0.01	[−0.03, 0.04	0.34	.733
Gender male	−1.04	[−1.83, −0.25]	−2.59	.010
Perceived vaccine efficacy	1.49	[1.09, 1.89]	7.38	<.001
Flu vaccine efficacy guess	−0.01	[−0.03, 0.02]	−0.72	.474

Note

Adjusted *R* ^2^ = 0.26.

### Other analyses

We pre‐registered two other analyses. The first was a one‐tailed *t*‐test to test the hypothesis that participants who are given no information about flu vaccine efficacy would overestimate the true efficacy; for the purpose of this test, we used a conservative reference value of 60%. This hypothesis was supported, *t*(199) = 10.55, *p *< .001, with the mean efficacy estimate being 74%.

Our final pre‐registered test was of the correlation between estimated flu vaccine efficacy (for those in the no information conditions) and stated flu vaccine intention. The observed correlation of 0.21 was significantly different from zero, *t*(198) = 3.03, *p *< .001.

## Discussion

The results of this study may have important implications for messaging strategies to boost vaccine uptake. Although our main focus is on boosting COVID‐19 vaccine uptake, a better understanding of how efficacy information influences the public is relevant for other vaccination programmes, notably the annual flu vaccination programme.

### COVID vaccine intentions

There are three main findings from our study. The first is that the provision of safety and efficacy information works. Simply providing information about the safety and efficacy of the new COVID‐19 vaccines resulted in vaccination intentions that were, on average, 0.39 *SD* higher than those in the no information condition. We are not able to say whether efficacy or safety information was more important, but any appropriate messaging should include both forms of information, to address both response efficacy and response costs. The importance of PVE and safety is consistent with findings that have been published in the months since our paper was originally submitted. For example, Kaplan and Milstein (2021) report a study in which representative samples of the US population were presented with scenarios that varied vaccine efficacy and side effects; results showed that vaccine acceptance improved when the efficacy increased above 70%. The authors conclude that, ‘our results indicate that expected efficacy is the most important factor in the decision to accept a vaccine’ (p. 5). In a study that involved cross‐sectional structured interviews with 1,427 elderly people living in Poland, concerns about the efficacy of the vaccine or its potential side‐effects were the most frequent justifications among those who chose not to be vaccinated (Malesza & Bozym, 2021). Similarly, Han et al. (2021) found that lack of confidence in the efficacy and safety of COVID‐19 vaccines were the most important reasons for vaccine hesitancy among the migrant population in Shanghai.

The second main finding from our study is that providing COVID vaccine efficacy information in the context of information about flu vaccine efficacy being much lower (40%) resulted in even stronger vaccination intentions; specifically, information about flu vaccine efficacy resulted in a further increase in intentions (beyond the provision of COVID‐19 vaccine information) of an additional 0.31 *SD*. This effect of context is what we predicted based on a positive contrast effect on the most salient dimension (Schwartz & Bless, 1992; Tversky, 1977). The impact of emphasizing vaccine efficacy further reaffirms the importance of the psychological dimension of response efficacy, in accord with protection motivation theory.

Another way in which the flu vaccine context may have helped is with respect to the processing of other less salient dimensions. The inclusion/exclusion model of Schwartz and Bless (1992) suggests that context effects may result in assimilation for some dimensions. That is, the juxtaposition of COVID‐19 and flu vaccines may have led to the perception that the two types of vaccines are similar on characteristics other than efficacy, such as safety. Assimilation to the characteristics of a more familiar, trusted vaccine is likely to have a beneficial effect on attitudes towards a novel vaccine. However, we did not directly measure perceptions of vaccine safety, so this interpretation is somewhat speculative.

### Flu vaccine intentions

The third main finding from our study is that the positive contrast effect for the COVID‐19 vaccine did not appear to be accompanied by a negative contrast effect on flu vaccine intentions. The utility of the messaging approach that we trialled here would be greatly reduced if providing information about the flu vaccine efficacy resulted in a reduction in the number of people receiving flu vaccinations. It is reassuring that our data do not show a negative impact of flu vaccine efficacy information on vaccine intentions. It is possible that a larger study with greater power might detect a small negative effect. Nevertheless, weighing this possible cost against the possible benefit of increasing vaccination against COVID‐19 – a far deadlier disease – we would argue that this is a risk that may be worth taking. Initial indications are that the Northern Hemisphere may follow the Southern Hemisphere in experiencing a relatively modest flu season this year. There will be opportunity to increase flu vaccine intentions next season, and the success of the COVID‐19 vaccination programme is likely to be an important influence on this outcome.

### Estimated objective efficacy versus perceived efficacy

As we anticipated, participants who were not given any information about the efficacy of the annual flu vaccine tended to overestimate its true efficacy: their mean estimate was 74%, whereas the typical efficacy is closer to 40%, and not higher than 60% in any recent year (Cohen, 2017). However, our data also suggest that the way in which individuals perceive the response efficacy of vaccines is not perfectly related to the percentage efficacy that is measured in clinical trials, and that perceived efficacy is the more important factor in determining intentions. Although there was a significant positive correlation between perceived flu vaccine efficacy and estimated vaccine efficacy, it was by no means perfect. To illustrate, among participants with the maximum possible perceived efficacy score (i.e., those who strongly agreed that the flu vaccine is effective in preventing the flu), the estimates of vaccine efficacy ranged between 65% and 100%. We might infer that, for many individuals, objective efficacies well below 100% are sufficient to endow a vaccine with a high level of psychological response efficacy. Likewise, in our regression analysis, perceived efficacy was a significant predictor of flu vaccine intentions, whereas estimated objective vaccine efficacy was not. This aspect of our results is consistent with the important role of perceived response efficacy in protection motivation theory.

### Limitations

A limitation of our study is that our participants are not a representative sample of the UK population, and it is conceivable that the impact of vaccine information would be different in the broader population. However, we note that the sample included participants drawn from all around the United Kingdom, with a wide range of (normally distributed) ages.

Another limitation of our results is that we measured intentions rather than actual vaccination behaviour. This reliance on hypothetical behaviour was unavoidable, given that the United Kingdom had only just begun COVID‐19 vaccination a few days before our data collection, and this vaccination was restricted to health care workers and those over 80. Nevertheless, previous research has found that an individual’s vaccination intention is a strong predictor of whether they go on to take a vaccine (e.g., DiBonaventura & Chapman, 2005; Godin, Vézina‐Im, & Naccache, 2010). Likewise, we did not directly measure how the provision of efficacy information changed vaccine confidence, although intentions may reasonably be considered a proxy for confidence.

One possible concern about the reliance on self‐reported intentions following an experimental treatment is that our design may be vulnerable to demand effects. However, this is unlikely to be a major concern, particularly for the comparative efficacy effect. We employed a between‐subjects design, which is more conservative and less susceptible to demand effects than a within‐subjects design (Charness, Gneezy & Kuhn, 2012). There is no reason why demand effects would be greater for participants who saw flu vaccine information in addition to information about the COVID‐19 vaccine. Even the overall vaccine information effect is unlikely to reflect demand effects. Recent research indicates that online survey experiments are robust to experimenter demand. Mummolo & Peterson, (2019) replicated a range of experimental designs and showed that providing participants with information about experimenter expectations did not alter the treatment effects – indeed, even financial incentives to respond in accordance with these expectations failed to consistently induce demand effects.

### Applications

We believe that the comparative efficacy approach that we tested here could be applied in real‐world messaging campaigns, and that doing so may help to tackle the problem of vaccine hesitancy. The size of the effect that we observed was not large, but it is important to note that our intervention was a single‐shot message consisting of 200 words, which most participants read in less than a minute. In practice, a public messaging campaign would present vaccine‐hesitant individuals with this message on multiple occasions. Our expectation is that this would result in a stronger effect, and that many fencesitters would be encouraged to take up the offer of vaccination. The source of information is likely to be important: Betsch and Sachse (2013) reported experiments showing a backfire effect, whereby strong statements that vaccines do not cause risk led to increased concerns about vaccine harm when the source of the information was a pharmaceutical company.

It may be appropriate to target such campaigns at vulnerable populations where lower levels of vaccine confidence have resulted in lower vaccination rates. In the United States, Grumbach et al. (2021) found that Black, Latinx, and Asian respondents were approximately twice as likely as White respondents to express concerns about vaccine efficacy, and a similar hesitancy has been observed in the BAME community in the United Kingdom (Iyengar, Vaishya, Jain, & Ish, 2021). Globally, there have been reports of low rates of COVID‐19 vaccine acceptance in the Middle East, Russia, and Africa (Sallam, 2021). It is possible to envisage a modified comparative efficacy message being used in African countries in which mass vaccination programmes in recent years have eliminated polio and virtually eliminated meningitis due to type A meningococcus.

A potential drawback that might be considered concerns the possibility of discouraging uptake of lower efficacy vaccines. In principle, messaging that emphasizes the very high efficacy of certain COVID‐19 vaccines could be damaging to the uptake of COVID‐19 vaccines with lower levels of efficacy. The UK vaccination programme has relied heavily on the Oxford‐AstraZeneca vaccine, which had not yet completed clinical trials at the time of our experiment. The final outcome of these trials showed an efficacy against symptomatic disease of 74.2%, that is, higher than the flu vaccine but not as high as those of the two COVID‐19 vaccines to which we referred. Nevertheless, mass vaccination data show that a single dose of the Oxford–AstraZeneca vaccine was associated with a vaccine effect of 94% against hospital admission for COVID‐19‐related hospitalization (Vasileiou et al., 2021), and thus it would still be possible to cite very high efficacy data for this vaccine.

More broadly, our study illustrates a comparative efficacy messaging approach that is not tied to the specific vaccines that we referred to, but could be used in any situation where a large contrast between vaccine efficacy can be exploited. In theory, if a nation had access to two vaccines, one of which was of much lower efficacy than the other, the type of messaging that we propose might not be appropriate, and the national health service might prefer different forms of messaging. Even in this hypothetical situation, though, the comparative efficacy messaging approach might be a good option to consider, especially if it were feasible to hold back doses of the higher efficacy vaccine that could then be offered to more hesitant members of the population.

### Conclusion

The rapid development of several COVID‐19 vaccines is a noteworthy achievement for modern medicine. The public health focus has shifted to the successful distribution of the vaccine and the effective messaging that will ensure a high uptake. Drawing on psychological theory enables us to better understand factors that influence vaccine intentions and behaviour. This perspective suggests a focus on developing messages that boost response efficacy and reduce perceived response costs. The approach demonstrated here shows how vaccination intentions can be strengthened through a simple messaging intervention that utilizes context effects to increase perceived response efficacy.

References

Bednarczyk, R. A.
, 
Chu, S. L.
, 
Sickler, H.
, 
Shaw, J.
, 
Nadeau, J. A.
, & 
McNutt, L.‐A.
 (2015). Low uptake of influenza vaccine among university students: Evaluating predictors beyond cost and safety concerns. Vaccine, 33(14), 1659–1663.2572832010.1016/j.vaccine.2015.02.033

Betsch, C.
, & 
Sachse, K.
 (2013). Debunking vaccination myths: Strong risk negations can increase perceived vaccination risks. Health Psychology, 32(2), 146.2240926410.1037/a0027387

Bish, A.
, 
Yardley, L.
, 
Nicoll, A.
, & 
Michie, S.
 (2011). Factors associated with uptake of vaccination against pandemic influenza: A systematic review. Vaccine, 29(38), 6472–6484.2175696010.1016/j.vaccine.2011.06.107

Brewer, N. T.
, 
Chapman, G. B.
, 
Gibbons, F. X.
, 
Gerrard, M.
, 
McCaul, K. D.
, & 
Weinstein, N. D.
 (2007). Meta‐analysis of the relationship between risk perception and health behavior: The example of vaccination. Health Psychology, 26(2), 136.1738596410.1037/0278-6133.26.2.136

Brewer, N. T.
, 
Chapman, G. B.
, 
Rothman, A. J.
, 
Leask, J.
, & 
Kempe, A.
 (2017). Increasing vaccination: Putting psychological science into action. Psychological Science in the Public Interest, 18(3), 149–207.2961145510.1177/1529100618760521

Camerini, A.‐L.
, 
Diviani, N.
, 
Fadda, M.
, & 
Schulz, P. J.
 (2019). Using protection motivation theory to predict intention to adhere to official MMR vaccination recommendations in Switzerland. SSM‐Population Health, 7, 100321.10.1016/j.ssmph.2018.11.005PMC629308030581956
CDC
(2018). Estimates of Flu Vaccination Coverage among Children – United States, 2017–18 Flu Season. Retrieved from https://www.cdc.gov/flu/fluvaxview/coverage‐1718estimates.htm

CDC
. (2019). Past seasons vaccine effectiveness estimates. Retrieved from https://www.cdc.gov/flu/vaccines‐work/past‐seasonsestimates.html


Charness, G.
, 
Gneezy, U.
, & 
Kuhn, M. A.
 (2012). Experimental methods: Between‐subject and within‐subject design. Journal of economic behavior & organization, 81(1), 1–8.

Chapman, G. B.
, & 
Coups, E. J.
 (1999). Predictors of influenza vaccine acceptance among healthy adults. Preventive Medicine, 29(4), 249–262.1054705010.1006/pmed.1999.0535

Cohen, J.
 (2017). Why is the flu vaccine so mediocre?
Science, 357(6357), 1222–1223.2893578310.1126/science.357.6357.1222

Detoc, M.
, 
Bruel, S.
, 
Frappe, P.
, 
Tardy, B.
, 
Botelho‐Nevers, E.
, & 
Gagneux‐Brunon, A.
 (2020). Intention to participate in a covid‐19 vaccine clinical trial and to get vaccinated against covid‐19 in France during the pandemic. Vaccine, 38(45), 7002–7006.3298868810.1016/j.vaccine.2020.09.041PMC7498238

DiBonaventura, M. D.
, & 
Chapman, G. B.
 (2005). Moderators of the intention–behavior relationship in influenza vaccinations: Intention stability and unforeseen barriers. Psychology and Health, 20(6), 761–774.

Dryhurst, S.
, 
Schneider, C. R.
, 
Kerr, J.
, 
Freeman, A. L. J.
, 
Recchia, G.
, 
van der Bles, A. M.
, … 
van der Linden, S.
 (2020). Risk perceptions of covid‐19 around the world. Journal of Risk Research, 23(7‐8), 994–1006.

Ehrenstein, B. P.
, 
Hanses, F.
, 
Blaas, S.
, 
Mandraka, F.
, 
Audebert, F.
, & 
Salzberger, B.
 (2010). Perceived risks of adverse effects and influenza vaccination: A survey of hospital employees. European Journal of Public Health, 20(5), 495–499.2008967710.1093/eurpub/ckp227

Ernsting, A.
, 
Lippke, S.
, 
Schwarzer, R.
, & 
Schneider, M.
 (2011). Who participates in seasonal influenza vaccination? Past behavior moderates the prediction of adherence. Advances in Preventive Medicine, 2011, 148934.2199143010.4061/2011/148934PMC3168914

Flynn, M.
, & 
Ogden, J.
 (2004). Predicting uptake of MMR vaccination: A prospective questionnaire study. British Journal of General Practice, 54(504), 526–530.PMC132480515239915

Freimuth, V. S.
, 
Jamison, A. M.
, 
An, J.
, 
Hancock, G. R.
, & 
Quinn, S. C.
 (2017). Determinants of trust in the flu vaccine for African Americans and Whites. Social Science & Medicine, 193, 70–79.2902855810.1016/j.socscimed.2017.10.001PMC5706780

Godin, G.
, 
Vézina‐Im, L.‐A.
, & 
Naccache, H.
 (2010). Determinants of influenza vaccination among healthcare workers. Infection Control & Hospital Epidemiology, 31(7), 689–693.2048237310.1086/653614

Grumbach, K.
, 
Judson, T.
, 
Desai, M.
, 
Jain, V.
, 
Lindan, C.
, 
Doernberg, S. B.
, & 
Holubar, M.
 (2021). Association of race/ethnicity with likeliness of covid‐19 vaccine uptake among health workers and the general population in the San Francisco bay area. JAMA. Internal Medicine, Published Online First: 30 March 2021 e211445. https://jamanetwork.com/journals/jamainternalmedicine/article‐abstract/2778176
10.1001/jamainternmed.2021.1445PMC801064333783471

Gust, D.
, 
Brown, C.
, 
Sheedy, K.
, 
Hibbs, B.
, 
Weaver, D.
, & 
Nowak, G.
 (2005). Immunization attitudes and beliefs among parents: Beyond a dichotomous perspective. American Journal of Health Behavior, 29(1), 81–92.1560405210.5993/ajhb.29.1.7

Gust, D. A.
, 
Strine, T. W.
, 
Maurice, E.
, 
Smith, P.
, 
Yusuf, H.
, 
Wilkinson, M.
, … 
Schwartz, B.
 (2004). Underimmunization among children: Effects of vaccine safety concerns on immunization status. Pediatrics, 114(1), e16–e22.1523196810.1542/peds.114.1.e16

Han, K.
, 
Francis, M. R.
, 
Zhang, R.
, 
Wang, Q.
, 
Xia, A.
, 
Lu, L.
, … 
Hou, Z.
 (2021). Confidence, acceptance and willingness to pay for the covid‐19 vaccine among migrants in shanghai, china: A cross‐sectional study. Vaccines, 9(5), 443.3406318210.3390/vaccines9050443PMC8147504

Iyengar, K. P.
, 
Vaishya, R.
, 
Jain, V. K.
, & 
Ish, P.
 (2021). BAME community hesitancy in the UK for covid‐19 vaccine: Suggested solutions. Postgraduate Medical Journal. Published Online First: 29 March 2021. 10.1136/postgradmedj-2021-139957
35232873

Kaplan, R. M.
, & 
Milstein, A.
 (2021). Influence of a COVID‐19 vaccine’s effectiveness and safety profile on vaccination acceptance. Proceedings of the National Academy of Sciences, 118(10), e2021726118.10.1073/pnas.2021726118PMC795819233619178

Kim, S.
, 
Pjesivac, I.
, & 
Jin, Y.
 (2019). Effects of message framing on influenza vaccination: Understanding the role of risk disclosure, perceived vaccine efficacy, and felt ambivalence. Health Communication, 34(1), 21–30.2905336910.1080/10410236.2017.1384353

Kwok, K. O.
, 
Lai, F.
, 
Wei, W. I.
, 
Wong, S. Y. S.
, & 
Tang, J. W.
 (2020). Herd immunity–estimating the level required to halt the covid‐19 epidemics in affected countries. Journal of Infection, 80(6), e32–e33.3220938310.1016/j.jinf.2020.03.027PMC7151357

Lewandowsky, S.
, 
Ecker, U. K.
, 
Seifert, C. M.
, 
Schwarz, N.
, & 
Cook, J.
 (2012). Misinformation and its correction: Continued influence and successful debiasing. Psychological Science in the Public Interest, 13(3), 106–131.2617328610.1177/1529100612451018

Ling, M.
, 
Kothe, E. J.
, & 
Mullan, B. A.
 (2019). Predicting intention to receive a seasonal influenza vaccination using protection motivation theory. Social Science & Medicine, 233, 87–92.3119519410.1016/j.socscimed.2019.06.002

Maddux, J. E.
, & 
Rogers, R. W.
 (1983). Protection motivation and self‐efficacy: A revised theory of fear appeals and attitude change. Journal of Experimental Social Psychology, 19(5), 469–479.

Malesza, M.
, & 
Bozym, M.
 (2021). Factors influencing covid‐19 vaccination uptake in an elderly sample in Poland. medRxiv 2021.03.21.21254047. 10.1101/2021.03.21.21254047

Moderna‐Press‐Release
(2020). Moderna Announces Primary Efficacy Analysis in Phase 3 Cove Study for its Covid‐19 Vaccine Candidate. Retrieved from https://investors.modernatx.com/news‐releases/news‐release‐details/moderna‐announces‐primary‐efficacy‐analysis‐phase‐3‐cove‐study


Mummolo, J.
, & 
Peterson, E.
 (2019). Demand effects in survey experiments: An empirical assessment. American Political Science Review, 113(2), 517–529.

Murphy, J.
, 
Vallières, F.
, 
Bentall, R. P.
, 
Shevlin, M.
, 
McBride, O.
, 
Hartman, T. K.
, … 
Hyland, P.
 (2021). Psychological characteristics associated with covid‐19 vaccine hesitancy and resistance in Ireland and the United Kingdom. Nature Communications, 12(1), 1–15.10.1038/s41467-020-20226-9PMC778269233397962

Ozawa, S.
, 
Yemeke, T. T.
, 
Evans, D. R.
, 
Pallas, S. E.
, 
Wallace, A. S.
, & 
Lee, B. Y.
 (2019). Defining hard‐to‐reach populations for vaccination. Vaccine, 37(37), 5525–5534.3140091010.1016/j.vaccine.2019.06.081PMC10414189

Pareek, M.
, & 
Pattison, H. M.
 (2000). The two‐dose measles, mumps, and rubella (MMR) immunisation schedule: Factors affecting maternal intention to vaccinate. British Journal of General Practice, 50(461), 969–971.PMC131388311224968
Pfizer‐Press‐Release
(2020). Pfizer and Biontech Conclude Phase 3 Study of Covid‐19 Vaccine Candidate, Meeting All Primary Efficacy Endpoints. Retrieved from https://www.pfizer.com/news/press‐release/press‐release‐detail/pfizer‐and‐biontech‐conclude‐phase‐3‐study‐covid‐19‐vaccine


Pierre, J. M.
 (2020). Mistrust and misinformation: A two‐component, socio‐epistemic model of belief in conspiracy theories. Journal of Social and Political Psychology, 8(2), 617–641.

Quinn, S.
, 
Jamison, A.
, 
Musa, D.
, 
Hilyard, K.
, & 
Freimuth, V.
 (2016). Exploring the continuum of vaccine hesitancy between African American and White adults: Results of a qualitative study. PLoS Currents, 8. 10.1371/currents.outbreaks.3e4a5ea39d8620494e2a2c874a3c4201
PMC530912328239512

Rosselli, R.
, 
Martini, M.
, 
Bragazzi, N. L.
, 
Watad, A.
, & Fluad Effect Working Group
(2017). The public health impact of the so‐called “fluad effect” on the 2014/2015 influenza vaccination campaign in Italy: Ethical implications for health‐care workers and health communication practitioners. In 

G.
Donelli

 (ed.), Advances in microbiology, infectious diseases and public health (pp. 125–134). Advances in Experimental Medicine andBiology, vol 973. Cham, CH: Springer. https://doi.org/10.1007/5584_2017_3910.1007/5584_2017_3928452003

Sah, P.
, 
Medlock, J.
, 
Fitzpatrick, M. C.
, 
Singer, B. H.
, & 
Galvani, A. P.
 (2018). Optimizing the impact of low‐efficacy influenza vaccines. Proceedings of the National Academy of Sciences, 115(20), 5151–5156.10.1073/pnas.1802479115PMC596032729712866

Sallam, M.
 (2021). COVID‐19 vaccine hesitancy worldwide: A concise systematic review of vaccine acceptance rates. Vaccines, 9(2), 160.3366944110.3390/vaccines9020160PMC7920465

Salmon, D. A.
, & 
Dudley, M. Z.
 (2020). It is time to get serious about vaccine confidence. The Lancet, 396(10255), 870–871.10.1016/S0140-6736(20)31603-232919522

Schwartz, N.
, & 
Bless, H.
 (1992). Constructing reality and its alternatives: An inclusion/exclusion model of assimilation and contrast effects in social judgments. In 

L.
Martin

 & 

A.
Tesser

 (Eds.), The construction of social judgments (pp. 217–245). Hillsdale, NJ: Lawrence Erbaum.

Sherman, S. M.
, 
Smith, L. E.
, 
Sim, J.
, 
Amlôt, R.
, 
Cutts, M.
, 
Dasch, H.
, … 
Sevdalis, N.
 (2020). COVID‐19 vaccination intention in the UK: Results from the covid‐19 vaccination acceptability study (CoVAccS), a nationally representative cross‐sectional survey. Human Vaccines and Immunotherapeutics, 17(6), 1612–1621.3324238610.1080/21645515.2020.1846397PMC8115754

Tversky, A.
 (1977). Features of similarity. Psychological Review, 84(4), 327.

Vasileiou, E.
, 
Simpson, C. R.
, 
Robertson, C.
, 
Shi, T.
, 
Kerr, S.
, 
Agrawal, U.
 (2021). Effectiveness of first dose of covid‐19 vaccines against hospital admissions in Scotland: National prospective cohort study of 5.4 million people. Available at SSRN: https://ssrn.com/abstract=3789264

WHO
(2014). Report of the Sage Working Group on Vaccine Hesitancy. Retrieved from http://www.who.int/immunization/sage/meetings/2014/october/SAGE_working_group_revised_report_vaccine_hesitancy.pdf


Witte, K.
, 
Meyer, G.
, & 
Martell, D.
 (2001). Effective health risk messages. A step‐by‐step guide. Thousand Oaks, CA: Sage.

Yeung, M. P.
, 
Lam, F. L.
, & 
Coker, R.
 (2016). Factors associated with the uptake of seasonal influenza vaccination in adults: A systematic review. Journal of Public Health, 38(4), 746–753.2815855010.1093/pubmed/fdv194

## Footnotes

^1^
The minimum proportion of the population needed to be immunized to achieve herd immunity is 1 ‐ 1/*R*_0_, where *R*_0_ is the reproduction number.

^2^
Our pre‐registration stated that we would aim to recruit 150 participants per condition, or the largest sample that research funds would allow. Given the greater than expected expense of pre‐screening to find participants who met our inclusion criteria, and the greater than anticipated number of participants who reported having had COVID‐19 (and therefore had to be excluded) we were unable to achieve this goal, and opted to stop when we had 100 participants per condition; we did not analyse the data until all data were collected.

## Information text presented in experiment

All participants except those in the no information condition saw the following text:

Many COVID‐19 vaccines are in development, and some of these vaccines have already completed ‘Phase 3’ trials in which thousands of human patients have been given the vaccine.

The results from the trials reported so far indicate that the vaccines are safe, with no serious side effects. The most severe side effects reported were fatigue (4%) and headache (2%). Both of these effects resolved shortly after vaccination.

Results from the trials reported so far also show that the COVID‐19 vaccines have very high levels of efficacy, meaning that they are very effective in protecting people against infection.

The final analysis of the Pfizer vaccine has reported an efficacy rate of 95%.

Final results from the trials of the Moderna vaccine confirm it has 94% efficacy.

Nobody who was vaccinated with either of these vaccines developed severe disease.

## Participants in the flu info – 40% efficacy condition then saw the following text:

There has never been a ‘100% effective’ vaccine, but these efficacy rates are not far off. By comparison, the average efficacy of the annual flu vaccine is 40%. Nevertheless the flu vaccine has prevented millions of infections and saved many thousand lives.

If we see a high uptake of COVID‐19 vaccination, the result will be a massive reduction in the number of COVID‐19 cases, hospitalizations, and deaths. This in turn will greatly reduce health care costs and ensure that our NHS is not overloaded.

## Participants in the flu info – 60% efficacy condition saw the following text:

There has never been a ‘100% effective’ vaccine, but these efficacy rates are not far off. By comparison, the efficacy of the annual flu vaccine has not been more than about 60% in recent years. Nevertheless the flu vaccine has prevented millions of infections and saved many thousand lives.

If we see a high uptake of COVID‐19 vaccination the result will be a massive reduction in the number of COVID‐19 cases, hospitalizations, and deaths. This in turn will greatly reduce health care costs and ensure that our NHS is not overloaded.

## Participants in the COVID‐19 vaccine info only condition saw the following text:

If we see a high uptake of COVID‐19 vaccination the result will be a massive reduction in the number of COVID‐19 cases, hospitalizations, and deaths. This in turn will greatly reduce health care costs and ensure that our NHS is not overloaded.
